# How Do Stress Situations Affect Higher-Level Text Processing in L1 and L2 Readers? An Eye-Tracking Study

**DOI:** 10.3390/jemr18020007

**Published:** 2025-03-24

**Authors:** Ziqing Xia, Chun-Hsien Chen, Jo-Yu Kuo, Mingmin Zhang

**Affiliations:** 1 School of Mechanical and Aerospace Engineering, Nanyang Technological University, Singapore 639798, Singapore; ziqing001@e.ntu.edu.sg (Z.X.); mchchen@ntu.edu.sg (C.-H.C.); 2 Department of Industrial Design, National Taipei University of Technology, Taipei 10608 City, Taiwan; 3 Institute of Cognitive Neuroscience, School of Psychology and Cognitive Science, East China Normal University, Shanghai 200050, China; keithmiles.ming@outlook.com

**Keywords:** eye-tracking, stress, reading comprehension, text processing, online learning

## Abstract

Existing studies have revealed that the reading comprehension ability of readers can be adversely affected by their psychosocial stress. Yet, the detailed impact of stress on various stages of text processing is understudied. This study aims to explore how the higher-level text processing ability, including syntactic parsing, sentence integration, and global text processing, of first language (L1) and second language (L2) English readers is affected under stress situations. In addition, the roles of trait anxiety, the central executive function moderating stress effects, in text processing were also examined. Twenty-two L1 readers and twenty-one L2 readers were asked to perform reading comprehension tasks under different stress situations. Eye-tracking technology was adopted to record participants’ visual behaviors while reading, and ten eye-movement measurements were computed to represent the effect of different types of text processing. The results demonstrate that the stress reduced the efficiency of syntactic parsing and sentence integration in both L1 and L2 groups, but only impaired global text processing in L2 readers. Specifically, L2 readers focused more on the topic structure of text to facilitate comprehension under stress situations. Moreover, only L1 readers’ higher-level text processing was affected by trait anxiety, while L2 readers’ processing was mainly related to their reading proficiency level. Future studies and applications were discussed. The findings advance our understanding of stress effects on different stages of higher-level text processing. They also have practical implications for developing interventions to help language learners suffering from stress disorders.

## 1. Introduction

Stress is prevalent in academic settings, with nearly 20% of students experiencing severe stress, particularly during high-stakes assessments [1]. It impairs cognitive functions, including attention, memory, and problem-solving, leading to lower academic performance [2,3]. Chronic stress is also linked to anxiety, reduced self-efficacy, and disengagement from learning, potentially resulting in burnout and long-term academic decline. Addressing stress is crucial for enhancing student well-being and educational outcomes [4].

Reading comprehension, a fundamental component of the learning process, involves extracting meaning from text to understand and interpret information [5]. However, stress situations, such as examinations, can significantly impair reading performance, leading individuals to misinterpret texts, overlook critical information, or fail to constructively comprehend the textual content [6,7,8,9]. The situation becomes more complex and challenging for users with English as a second language (L2), who often exhibit poor reading performance under stress conditions [10,11]. This prevalent issue has garnered the attention of English teachers and educators worldwide, making it vital to delve into a comprehensive understanding of the effect stress has on reading. This understanding is crucial in the development of targeted teaching strategies and specialized training designed to enhance reading comprehension among students, particularly those struggling with English as a second language.

## 2. Literature Review

### 2.1. The Attentional Control Theory (ACT)

The attentional control theory (ACT) provides a useful framework to explain how stress affects cognitive performance [12]. For a better understanding of the ACT and the present work, it is important to distinguish between stress and anxiety. Stress is defined as a real or interpreted threat to the physiological or psychological integrity of an individual that results in physiological and/or behavioral responses [13]. Under the stress concept presented by McEwen [13], the term ‘stress’ can be considered as objective stimulus properties of a particular situation. The objectively stressful condition might not be perceived as threatening in some cases (e.g., when the person has the experience to cope with the situation).

Anxiety is defined as the anticipation of a future threat, and it can be either a trait or a state [14]. State anxiety is an acute response to a potential threat and a state of hypervigilance triggered by stress [15]. By contrast, trait anxiety is a relatively stable aspect of a person’s personality. According to the interaction model of anxiety, stress, and coping processes, it is the interaction between personal variables (e.g., trait anxiety, vulnerability) and stress situations (e.g., social threat, test) that induce a perception of threat, causing increases in state anxiety and corresponding reactions [16]. In a nutshell, state anxiety can be considered as an emotional response interactively triggered by trait anxiety and by stress situations [17].

According to the ACT, anxiety increases the allocation of attention to threat-related stimuli and task-irrelevant distracters, leaving insufficient processing resources available to the central executive of working memory, thereby affecting cognitive processing. Such an adverse effect of anxiety on performance becomes greater when overall task demands increase or under stress situations [12]. The ACT emphasizes an important distinction between performance effectiveness and processing efficiency. Effectiveness refers to the quality of task performance indexed by standard behavioral measures (generally, response accuracy). In contrast, efficiency refers to the relationship between the effectiveness of performance and the effort or resources spent in task performance, with efficiency decreasing as more resources are invested to attain a given performance level. Typically, anxiety impairs processing efficiency to a greater extent than performance effectiveness because people usually trade processing efficiency to maintain their effectiveness when they are allowed to use compensatory strategies (e.g., increased effort or increased use of processing resources).

### 2.2. The Effect of Stress on Reading

The ACT provides a framework to explain how people’s reading performance would be affected by anxiety. Reading is a complex process that requires a timely execution and the integration of a variety of cognitive, linguistic, and non-linguistic skills, ranging from the lower-level processing abilities involved in phonological awareness and word recognition to higher-level syntactic, semantic, text integration, and metacognition skills. Higher-level processing skills enable readers to integrate information across multiple sentences and paragraphs with their background knowledge to build a situational model of text [18,19,20]. The reading process relies heavily on the central executive component and the phonological loop component of working memory [21,22]. Given the important role of the central executive in the reading process, it is rational to assume that anxiety affects reading efficiency by depleting the resources available to the central executive of working memory.

According to the ACT, people who are high in state or trait anxiety tend to trade efficiency for effectiveness by increasing effort, especially under stress situations. There is empirical evidence to support these assumptions. Calvo and Carreiras [23] investigated the effect of trait anxiety on reading performance under stress situations (i.e., test conditions). They found that high-anxiety people scored comparably to the low-anxiety individuals but showed greater word-reading times. Both the increased eye movement and reading time show that high-anxiety people tended to exert more effort and made enhanced use of processing resources to compensate for the impaired reading performance, which is consistent with the hypotheses of the ACT.

Moreover, Calvo and his colleagues conducted several studies to investigate what compensation strategies people resort to in the reading context [24,25]. They found that reading regressions, increasing reading time, and articulatory rehearsal are the main auxiliary strategies used to compensate for the decreased functioning of the central executive under stress situations [24]. Of the three strategies, making regressions is the most predominant and preferred choice, followed by increasing reading time. [26] applied the eye-tracking technique to measure the participants’ regressive eye movements in the stress reading task and found that anxious participants spent more time on regressions and made larger regressions than non-anxious peers. However, the above-mentioned study did not identify the regression event types and the function of these regressions is not clear.

In more recent studies, language proficiency has been considered as an important factor that can moderate the effect of stress on reading performance. For example, Rai et al. [27] found that stress situations affected the efficiency of drawing complex inferences from extended text in non-fluent second language (L2) readers. Tsai and Li [28] investigated how trait anxiety (trait test anxiety and trait foreign language reading anxiety) affect foreign language reading performance in the test conditions. They found that reading proficiency was negatively related to test anxiety and foreign language reading anxiety. Rai et al. [10] examined the effect of psychosocial stress and task difficulties on reading performance among L1 and L2 readers. They found that stress only impaired the reading efficiency of L2 readers who are high in trait foreign language reading anxiety. Their studies showed that the effect of stress on reading performance varied across people, situations, and task demands. These studies highlighted the importance of considering language proficiency and trait anxiety when investigating the effects of stress on reading performance.

Relatively less attention was paid to investigating the effect of stress on specific component processes involved in text processing. Calvo and Carreiras [23] found that under stress situations, only higher-level processes including the sentence integration and construction of situational models of text were affected, rather than lower-level processes such as word encoding. This study used word-level, sentence-level, and text-level psycholinguistic attributes (e.g., word length, place in the sentence) as predictor variables to reflect various processes involved in text processing. However, the time-window method used in the study only allows subjects to read one word at a time and forbid them to look back to the previous text. The study did not record the reading process in a natural way and ignored the important role of regression (backward eye movement) in the reading process. In the study of Rai et al. [10], they used questions with different inferential complexity to examine stress’s effect on the surface level, text-based level, and situation model level of the memory representation of text. They found that stress only impaired L2 readers’ efficiency to build a situational model of text. However, this study only investigated the outcome of text processing at the memory level, but not the ongoing process of text processing. In a nutshell, the effect of stress on various stages of higher-level text processing, including syntactic parsing, sentence integration, and global text processing, have not been fully explored yet. And we lack a method which can reflect the natural and ongoing process of text processing.

Moreover, to the authors’ knowledge, no previous study has investigated the effect of stress on topic structure processing. A typical expository text usually has a hierarchical structure developed surrounding a global topic. Proficient readers seek to identify and use the topic structure of text to organize their own understanding, which is an efficient strategy to reduce memory demands. Sensitivity to the topic structure can reflect readers’ higher-level processing skills like meta-cognition ability. It is still not known whether readers would change their strategy for processing topic structure under stress situations.

### 2.3. Research Questions

The primary objective of the present study is to explore how stress situations affect higher-level text processing including syntactic parsing, sentence integration, and global text processing in L1 and L2 readers. To be more specific, the following research questions were investigated in this study:

- Q1: How do stress situations affect higher-level text processing including syntactic parsing, sentence integration, and global processing in L1 and L2 readers?
- Q2: Do readers change their strategies for topic structure processing under stress situations?
- Q3: How do trait anxiety and the central executive function interact with stress situations in affecting higher-level text processing?

According to the ACT and findings from previous studies, the following was hypothesized:

- H1: There are greater adverse effects of stress on L2 than L1 readers in terms of their higher-level text processing, including syntactic parsing, sentence integration, and global text processing.
- H2: People tend to utilize the topic structure processing strategy to facilitate comprehension under stress situations.
- H3: Stress has a greater adverse effect on higher-level text processing among people with higher trait anxiety and in people with lower central executive functioning.

In order to record the natural and ongoing process of text processing, the eye-tracking technique is adopted in this study. It has been extensively utilized in reading research to understand the text processing effects and cognitive strategies of readers [29,30,31,32,33,34,35]. A large number of eye-movement measures have been designed to index different text processing stages, ranging from lexical access (to process words) to syntactic parsing (to process sentences) and global text processing (to integrate information from sentences that are not adjacent in the text) [36]. In the present study, various eye-movement measures will be calculated to index the various processes involved in text processing. The eye-movement measures will serve as a medium to study the effect of stress on higher-level text processing. The details of the eye-movement measures will be introduced in a later section.

## 3. Method

### 3.1. Participants

For recruitment purposes, an advertisement appealing to volunteers was posted as leaflets within the premises of Nanyang Technological University. G*Power [37] was used to calculate the a priori sample size, and the results indicated that at least 36 subjects were needed for a power of 90% with an effect size setting of 0.25. This research was approved by the Institutional Review Board of Nanyang Technological University [IRB-2021-320].

Participants were selected according to their self-reported English Proficiency level and English test score to ensure they have sufficient English proficiency to complete the reading tasks. A total of 44 students from [masked for blind review] were recruited for this study. One participant dropped out of the experiment, leaving a usable sample size of 43 (i.e., 23 females and 20 males, ages from 20 to 31 years old, with a mean (M) of 24.19 years and standard deviation (SD) of 2.41). Out of the 43 participants, 22 were first language (L1) English speakers, while the remaining 21 were second language (L2) English speakers, all of whom had Mandarin Chinese as their mother tongue. All L2 participants had valid IEFLTS or TOEFL test scores, with 18 of them having taken the IELTS test (M score = 6.79, SD = 0.50) and 3 of them having taken the TOEFL test (M score = 95.33, SD = 1.53). According to the score conversion standard provided by IELTS and TOEFL Official, candidates with an IELTS score of 6.79 and a TOEFL score of 95.33 are considered equally proficient in English. All participants self-reported their English reading proficiency level via the self-assessment tool established by the Association of Language Testers in Europe (ALTE) [38], and only participants who rated themselves as equal or above level 3 (i.e., can scan texts for relevant information and understand detailed instructions or advice) were recruited in the experiment. Previous studies have shown high correlations between self-rated language proficiency and objective proficiency measures, indicating the validity of using this measure in the present study [39,40].

### 3.2. Materials and Apparatus

As suggested by Rai et al. [10], the reading comprehension task should adopt challenging reading materials, such as the Graduate Record Examination (GRE), to induce a stronger effect of stress on L1 readers. This experiment utilized six short passages and their corresponding multiple-choice questions from the GRE Verbal Reasoning Practice. These materials were extracted from ‘GRE Reading White Paper’, a GRE study book featuring 170 original GRE passages, categorized by difficulty, structure, theme, and question type [41]. For this study, six medium-difficulty passages of equivalent length and structure were chosen. Considering the mechanical engineering background of all participants, the passages spanned various topics, including biology, culture, history, and others, aiming to minimize potential biases that might arise from varying levels of familiarity with the reading material’s subject matter.

The passages chosen were approximately the same length, with an average word count of around 158.5 words and a standard deviation of 8.38 words. The word count was carefully chosen to ensure that the text length would neither be too long to overwhelm nor too short to underserve the participants while still being sufficient for collecting substantial eye-movement data. Each passage followed a ‘general-to-specific’ structure, comprising a main idea and two supporting arguments, with the ‘topic-introducing sentence’ and the ‘viewpoint sentence’ being clearly marked in the book’s explanatory section.

**Figure 1:**
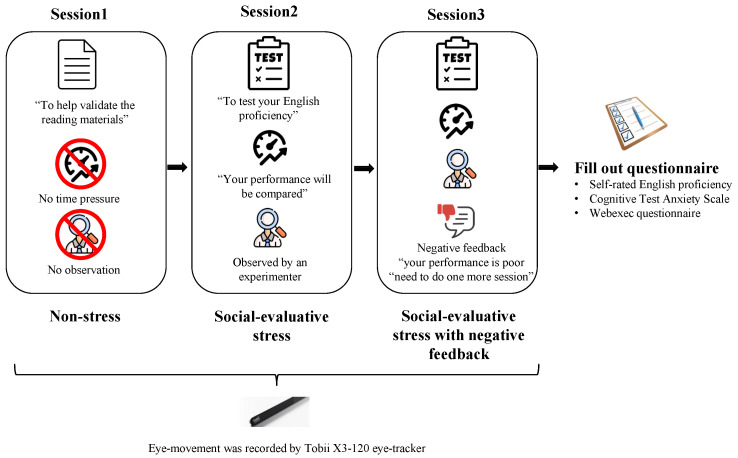
Illustration of experiment procedure.

**Figure 2:**
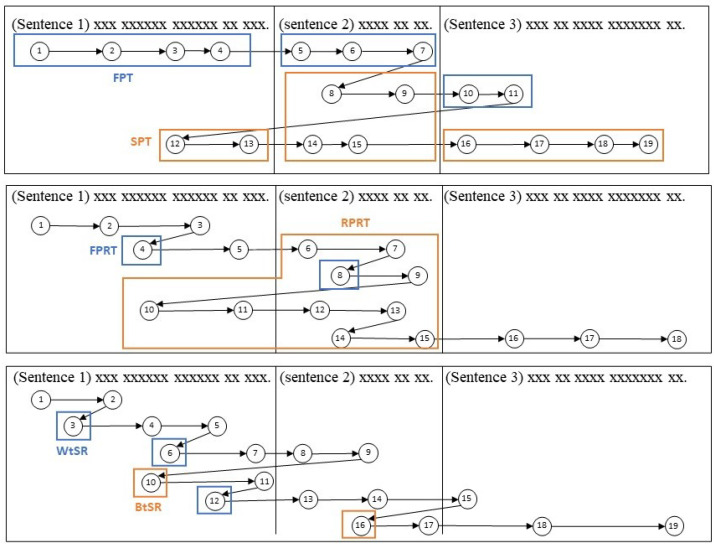
Illustration of eye-movement measures. (Note: the numbers represent fixations).

A 28-inch monitor with a resolution of 1920 × 1080 was used to display the reading materials. The reading task was administrated by the Tobii Studio Pro version 3.4.8 software. In the reading comprehension task, the passages were presented on the screen first. Each passage was presented in one paragraph and on one page, as shown in Figure 1. Following each text item, three multiple-choice questions were asked to assess the reading comprehension performance of participants. Each question was presented on one page, together with passage text, as shown in Figure 2. Readers were allowed to review the text when answering questions. All the text was displayed in Times New Roman font, size 12, which is a standard font choice in research due to its readability and ubiquity in academic and professional settings. In this study, the Tobii X3-120 eye tracker with a sampling rate of 120 Hz was utilized to track and record participants’ eye-movement data. The raw eye-movement data were exported from Tobii Studio Pro version 3.4.8. The eye-movement measures were processed with python 3.6.

### 3.3. Procedure

The whole experiment consisted of three sessions in a fixed order. In order to remove the variability caused by the presentation sequence of the reading materials, the current study adopted the Latin square design method to divide the six passages into three blocks of two passages each, with counterbalance order both within and between blocks. Stress was a within-participant factor, with every participant being in the non-stress condition for the first block of passages (session 1), in the social-evaluative stress condition for the second block of passages (session 2), and in the negative-feedback condition for the third block of passages (session 3), as shown in Figure 1. All participants followed a fixed order of stress conditions in three sessions to avoid any stress spill-over effects [42]. In each session, the participant read two passages and answered three multi-choice questions for each passage. Each participant was tested individually. After signing the consent form, the participant was presented with a sample reading task to familiarize them with the format, length, and difficulty of the texts and the types of questions. The sample task also helped reduce the stress effect caused by unfamiliar tasks. Both the on-screen and verbal instructions were given in English.

Session 1 presented a non-stress condition. The participants were told that the purpose of the tasks was not to rate their performance but to validate the reading materials, because “we did not know if the difficulty level of passage is appropriate”. To ensure participants fully comprehend the passage before answering questions, the experimenter emphasized the instruction “Please proceed to answer questions only after you have comprehended the passage” before the task starts. Also, the participants did not have the time pressure to perform the first reading task.

Session 2 presented the social-evaluative stress condition. The participants were told that reading performance will be evaluated regarding both the accuracy and reaction time they respond to the questions. To make sure participants read passages carefully and comprehend them in the first place, they were told that the reaction time will only be counted when they start answering questions and that there would be no time limit for them to read the passages. The participants were told that their reading performance will be compared with their peers’ performances and that the results will be given immediately after the test. Moreover, they were told that they had to undergo more sessions if they failed to reach an average performance. False expectations were given to participants by telling them that the average score was derived from a group of participants who obtained a good result on the GRE test. In addition to performance evaluation, two other stress manipulations were established in the presence of the experimenter and a video camera. The participants were informed that the entire test will be conducted under the surveillance of a video camera and that the video recordings will be subjected to further analysis, such as looking at cognitive performance, facial expressions, and body language. The experimenter sat beside the seated participant and looked at the participants. During the whole session, the experimenter kept a distant and strict attitude, pretending to keep taking notes about the reading performance of the participants.

Session 3 introduced the negative feedback to the stress condition. The participants were told that they “failed” to reach the average performance in session 2 and needed to complete one more session. Right before the reading task, participants received a brief stressful instruction, such as “Your reading performance is poor compared to others, you need to improve both your accuracy and reaction time”. Other instructions and stress manipulations were the same as in session 2.

After completing the three sessions, all participants were able to rest, they were debriefed on the deception of the stress manipulation, and small gifts were given for mood repair. In the end, they were asked to complete the Cognitive Test Anxiety Scale (CTAS) to assess the trait cognitive anxiety occurring in a cognitive test situation [43] and the webexec questionnaire to measure the executive control function [44].

### 3.4. Eye-Movement Measures

The eye-tracking technique can record visual cues of an ongoing reading task in real time in a non-intrusive way. In order to understand how readers process text under different stress situations, the present study calculated various eye-movement measures to reflect the various processes involved in higher-level text processing.

The syntactic parsing effect was reflected by the first-pass fixation time (FPT) and first-pass rereading time (FPRT) [45,46]. The FPT sums all fixation durations on the target sentence before exiting it, thereby offering insight into the reader’s processing efficiency upon first encounter with the sentence. The FPRT is computed from the total duration of all retrospective fixations on the target sentence during the first-pass reading, which indicates the ease or difficulty of lexical and syntactic processing when first approaching the sentence.

The sentence integration effect can be indexed by the regression path reading time (RPRT). This metric represents the time from the first fixation on the target sentence to when a fixation is made on the subsequent sentence [47,48]. This represents the process of a subject detecting a problem (e.g., syntactic ambiguity) and then rereading the prior area in order to build a concept. The second-pass fixation time (SPT), which is the total duration of all regressions back to the target sentence, reflects the process of integrating and consolidating sentence meanings to build a comprehensive text representation [45].

In summary, the syntactic processing effect was reflected by the first-pass fixation time (FPT) and first-pass rereading time (FPRT). The sentence integration effect was indexed by the regression path reading time (RPRT). The second-pass fixation time (SPT) denotes the process of re-analyzing the sentences to build a global representation of text, i.e., global text processing.

Topic structure processing is an essential part of global text processing. Readers who utilize topic structure to facilitate comprehension would selectively direct their attention to pertinent regions of the text, namely headings and topic sentences [45]. The topic structure processing effect can be indexed by the FPT and SPT spent on topic sentences [49]. Eye-movement measures that can represent topic structure processing were calculated to further investigate how readers process topic structure under different stress situations. Specifically, we focused on two types of sentences that indicate the structure of the passage, namely the topic-introducing sentence (TS), placed in a paragraph-initial position, and the viewpoint sentence (VS) that concludes the passage. Both the TS and VS can be referred to as topic sentences. The immediate and delayed effects of topic processing were examined by calculating the proportion of the FPT and SPT that belong to topic structure sentences. Table 1 summarized the description of each eye-movement measure used in this study. And Table 2 summarizes the mean and standard deviation of eye-movement measures under different stress conditions and language groups.

Moreover, previous studies have indicated that people tend to make more regressions in stress situations, but the type and function of these regressions are unknown. In the present study, this question will be addressed. The regressions made during text processing were divided into within-sentence regression (WtSR) and between-sentence regression (BtSR). WtSR can reflect the ease/difficulty of lexical processing. BtSR can reflect the process of switching between sentences to build the global representation of text, which is an important indicator of global text processing.

It is important to note that this study solely focused on participants’ eye movements during the solitary act of reading the passages, i.e., eye movements on the passage page. The experimental design required participants to fully comprehend the article before beginning to answer the questions, implying that the eye movements on the passage page encompassed the complete process of the participant handling a text. The entire dataset comprised 258 samples (43 participants × 6 passages). Eight eye-movement measures were derived from each sample’s raw eye-movement data. Among these measures, only BtSR was calculated based on the whole text; other measures were computed based on the unit of sentences—see Figure 2 for a simple diagram illustrating each of the measures. All sentence-level features’ values were computed as a ratio per line to adjust for differences in length across sentences. When examining the stress effect on text processing, the average values of sentence-level measures of all sentences, together with BtSR, were used as the dependent variables.

## 4. Results

### 4.1. Text Processing

A 2 (languages; between-subjects) × 3 (stress sessions; within-subject) repeated measures ANOVA was conducted on within-sentence regression (WtSR), between-sentence regression (BtSR), first-pass fixation time (FPT), second-pass fixation time (SPT), first-pass rereading time (FPRT), and regression path reading time (RPRT). The ANOVA results and effect sizes are summarized in Table 3. All statistical values have been corrected by the Greenhouse–Geisser approach. The significance level is set as 0.05.

**Figure 3:**
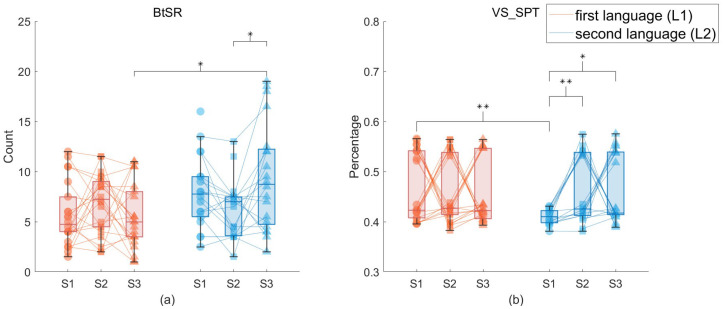
(**a**) Interaction effect between languages and stress for BtSR. (**b**) Interaction effect between languages and stress for VS_SPT sessions. S1 = session 1, S2 = session 2, S3 = session 3. * *p* < 0.05. ** *p*< 0.01. *** *p*< 0.001 level. L2 readers performed more between-sentence regressions than L1 readers in session 3 (t(36)=2.157,p=0.038,Cohen′sd=0.679). For L2 readers, they made more regressions in session 3 than in session 2, t(36)=−2.174,p=0.036,Cohen′sd=0.611. For L1 readers, there was no significant difference in BtSR under different sessions.

A medium-sized significant interaction effect between language and stress sessions was found for between-sentence regression (BtSR), $F_{\left(2,74\right)}=4.868,p=0.013,\eta^{2}=0.116$, as shown in Figure 3a.

There were large-sized statistically significant main effects of language on within-sentence regression (WtSR), first-pass fixation time (FPT), second-pass fixation time (SPT), first-pass rereading time (FPRT), and regression path reading time (RPRT). As shown in Table 4, L2 readers made significantly more within-sentence regression (WtSR) (t(40)=5.688,p<0.001) and used more first-pass fixation time (FPT) (t(41)=6.969,p<0.001), more second-pass fixation time (SPT) (t(38)=7.615,p<0.001), more first-pass rereading time (FPRT) (t(38)=4.526,p<0.001), and more regression path reading time (RPRT) (t(34)=7.140,p<0.001) than L1 readers. The main effect of stress conditions was found on first-pass rereading time (FPRT) $\left(F_{\left(2,37\right)}=5.667,p=0.006,\eta^{2}=0.130\right)$ and regression path reading time (RPRT) $\left(F_{\left(2,33\right)}=5.667,p=0.008,\eta^{2}=0.141\right)$. As shown in Table 5, both L1 and L2 readers spent more FPRT in session 2 than in session 1 (t(37)=3.170,p=0.003) and in session 3 (t(37)=2.554,p=0.015), and they spent less RPRT in session 1 than in session 2 (t(33)=−2.732,p=0.010) and session 3 (t(33)=−2.513,p=0.017).

To further examine the effects of stress, Helmert contrasts were conducted within the repeated measures ANOVA (Table 6). The results showed a significant overall effect of stress on FPRT (F(1,38)=5.174,p=0.029) and RPRT (F(1,34)=7.041,p=0.008), indicating that regression path reading time was generally higher under stress conditions compared to the baseline. Additionally, a significant difference between session 2 and session 3 was found for FPRT (F(1,38)=6.512,p=0.015), but not for RPRT (F(1,34)=0.437,p=0.513), suggesting that stress conditions affected first-pass rereading differently but did not significantly alter regression path reading time.

### 4.2. Topic Structure Processing

The repeated measures ANOVA method was used to examine the interaction effect of languages and stress sessions on TS_FPT, VS_FPT, TS_SPT, and VS_SPT. The ANOVA results and effect sizes are shown in Table 3. All statistical values have been corrected by the Greenhouse–Geisser approach.

There was a medium-sized languages × session interaction effect on VS_SPT (the ratio of second-pass fixation time on viewpoint sentences) $(F_{\left(2,72\right)}=3.405,p=0.043,$$\eta^{2}=0.086)$. In session 1, L1 group used more VS_SPT than L2 group, t(36)=3.353,p=0.002,Cohen′sd=1.152, as shown in Figure 3b. The VS_SPT spent by L2 readers significantly increased from non-stress to stress conditions, t(35)=2.800,p=0.007,Cohen′sd=1.421,t(35)=2.680,p=0.013,Cohen′sd=1.267).

Language showed large-sized statistically significant main effects on TS_FPT (ratio of first-pass fixation time on topic sentences) $(F_{\left(1,37\right)}=9.922,p=0.003,$$\eta^{2}=0.211)$ and TS_SPT (ratio of second-pass fixation time on topic sentences) $(F_{\left(1,41\right)}=9.428,$$p=0.004,\eta^{2}=0.187)$. More specifically, L1 spent more FPT (t(37)=3.091,p=0.003,Cohen′sd=0.704) and less SPT (t(41)=−4.000,p=0.004,Cohen′sd=0.122) on topic sentences than the L2 group.

### 4.3. Relationship Between Eye-Movement Measures, Personal Variables, and Reading Performance

In order to assess the relationships between personal variables (i.e., trait anxiety, central executive (CE), self-rated reading proficiency) and reading performance (i.e., reading accuracy and reading efficiency) and the variables of eye-movement measures, this study calculated their Pearson correlations for L1 and L2 readers, respectively. The results were shown in Table 7 and Table 8.

Rai et al. [10] emphasized that it is important to examine reading performance in terms of effectiveness and efficiency. Following Rai’s study, the effectiveness was indexed by response accuracy, which was calculated by the percentage of correct answers. The reading efficiency was obtained by dividing the accuracy by the responding time.

For L1 readers, as shown in Table 7, there were significant positive correlations between trait anxiety and text processing measures, including WtSR (r=0.440,p<0.001), FPT (r=0.313,p=0.011), SPT (r=0.299,p=0.015), FPRT (r=0.485,p<0.001), and RPRT (r=0.321,p=0.012). These findings suggest that L1 readers who had higher cognitive text anxiety tended to perform more within-sentence regression (WtSR) and use more first-pass fixation time (FPT), more second-pass fixation time (SPT), more first-pass rereading time (FPRT), and more regression path reading time (RPRT) when reading passages. To further explore the interaction effect between trait anxiety and stress on text processing, a median split on the trait anxiety scores was carried out, and a 3 (stress sessions) × 2 (high vs low trait anxiety) repeated ANOVA on the same eye-movement measures was performed. As a result, trait anxiety showed its main effect on L2 readers’ WtSR $\left(F_{\left(1,19\right)}=19.580,p<0.001,\eta^{2}=0.508\right)$, SPT $\left(F_{\left(1,24\right)}=5.600,p=0.033,\eta^{2}=0.286\right)$, and FPRT $\left(F_{\left(2,28\right)}=6.208,p=0.026,\eta^{2}=0.307\right)$, such that, compared to low-anxious individuals, high-anxious L2 readers demonstrated more WtSR (t(19)=4.424,p<0.001,Cohen′sd=1.531), more SPT (t(11)=1.386,p=0.033,Cohen′sd=1.061), and more FPT (t(11)=2.492,p=0.026,Cohen′sd=0.501). However, there was no significant interaction effect between stress conditions and trait anxiety. Moreover, there found a significant positive correlation between self-rated reading proficiency and TS_FPT (r=0.251,p=0.046), such that those L1 readers who rated themselves as having higher reading proficiency demonstrated higher FPT on TS.

As for the relationship between eye-movement measures and reading performance in L1 reading, there were significant positive correlations between BtSR and reading accuracy (r=0.337,p=0.006) and between BtSR and reading efficiency (r=0.279,p=0.024), such that those L1 readers who demonstrated more BtSR tended to have higher reading performance, both in terms of effectiveness and efficiency. Additionally, there was a significant negative correlation between reading accuracy and VS_SPT (r=−0.265,p=0.032) with L1 readers who spent less time on rereading and those who tended to have higher reading accuracy.

The correlations between eye-movement measures and personal variables were also analyzed for L2 readers, as shown in Table 8. The central executive was found to have significant negative correlations with three eye-movement measures, including SPT (r=−0.338,p=0.009), RPRT (r=−0.315,p=0.014), and VS_FPT (r=−0.274,p=0.036). These results suggest that L2 readers who reported having higher central executive problems used less SPT, less RPRT, and less VS_FPT.

The result also showed significant negative correlations between self-rated reading proficiency and three text processing measures, including WtSR (r=−0.297,p=0.018), BtSR (r=−0.289,p=0.028), and RPRT (r=−0.343,p=0.007). Those L2 readers who reported having higher reading proficiency tended to demonstrate less within-sentence regression (WtSR), less between-sentence regression (BtSR), and less regression path reading time (RPRT). Interestingly, a significant positive correlation was found between self-rated reading proficiency and CE among L2 readers (r=0.348,p=0.005). This suggests that L2 readers who rated themselves with high reading proficiency tended to have more executive problems.

For L2 readers, no significant correlation between reading performance and text processing measures was found. Nonetheless, topic structure processing measures were found to have correlations with reading performance in L2 reading. There was a significant positive correlation between accuracy and TS_SPT (r=0.258,p=0.041), such that those L2 readers who spent more time on rereading TS tended to have higher reading accuracy.

## 5. Discussion

### 5.1. How Stress Affects Higher-Level Text Processing (Q1)

The first question (Q1) sought to determine how stress situations affect higher-level text processing. According to the ACT, stress affects reading performance by impairing the central executive function, which is essential for processing texts [12]. Since stress primarily disrupts the goal-directed attentional system rather than the stimulus-driven attentional system, higher-level text processing, which relies more on goal-directed attention, is more susceptible to stress compared to lexical access, which is largely stimulus-driven. Previous research also supports this distinction, showing that anxiety primarily impairs sentence integration and the construction of situational models, whereas word-level processing remains largely unaffected [23]. For less proficient readers who lack the necessary skills for fluent word decoding, the cognitive effort required for lexical access and syntactic parsing may deplete central executive resources, leaving insufficient capacity for constructing a situational model of the text.

Moreover, prior studies have noted that readers would make more effort, such as increased reading time and regressions, to compensate for impaired processing effectiveness [23,24,25]. Thus, based on the literature review, we predicted greater effects of situational stress on L2 than L1 in terms of their higher-level text processing including syntactic parsing, sentence integration, and global text processing. It was hypothesized that L2 face more difficulties in syntactic processing, resulting in increased first-pass fixation time (FPT), first-pass rereading time (FPRT), and within-sentence regression (WtSR); sentence integration, resulting in increased regression path reading time (RPRT); and building global representations of text, resulting in increased second-pass fixation time (SPT) and between-sentence regression (BtSR) under stressful conditions.

The result of the current study was partially consistent with this hypothesis. The result showed that both L1 and L2 readers used more FPRT and RPRT under stressful conditions. This means that stress impaired the efficiency of syntactic parsing and sentence integration in both L1 and L2 groups. Even if the L1 group used much less FPRT and RPRT than the L2 group, indicating their higher-level proficiency in text processing than L2, their processing efficiency of syntactic parsing and sentence integration was still impaired under stress situations. Moreover, the result also supported the assumption of the ACT that individuals often compensate for impaired processing efficiency with additional effort when central executive resources are taxed. In the current reading experiment, the increased rereading time is the compensatory strategy used by readers to facilitate their impaired text processing.

While the stress impact on syntactic parsing and sentence integration was found in both L1 and L2 readers, the effect of stress on global text processing was only found in the L2 group. L2 readers showed increased BtSR when moving from non-stress to stressful sessions. This eye-movement measure represented the frequency of switching between sentences, which reflected the process of integrating and wrapping up the meaning of sentences to build global representations of text. The result indicates that L2 readers faced more difficulties in building the situational model of text under stressful conditions. To compensate for the impaired processing effectiveness, L2 readers kept switching between sentences to build the overall representation of the passage, while for the L1 group, their global text processing was not affected under stress situations.

### 5.2. How Stress Affects Topic Structure Processing (Q2)

The second research question (Q2) in this research was regarding the impact of stress on topic structure processing. Sentences that introduce or conclude discourse topics in an expository text usually place heavy processing demands on readers [45]. The efficient processing of topic structure sentences results in the greater facilitation of building the situational model of text. Prior studies have found that topic structure sentences were usually processed more slowly than other sentences when readers first encounter them [50,51]. Given that readers update a representation of the text’s topic structure each time they encounter topic sentences, this would speed up the rereading process of topic structure sentences if they have already constructed a topic structure representation during the first reading of a text [52]. Thus, those readers who are proficient at utilizing topic sentences to help construct global text representation would spend more FPT and less SPT on topic structure sentences.

Previous studies found that readers either rely on memory or making regressions to topic structure sentences when they want to review or access information associated with the topic [45]. And rereading topic structure sentences was an effective strategy to construct the global representation of text, especially when the reader consults memory [45]. Thus, it was hypothesized that stress would affect L2 readers’ global text processing and that L2 readers would use more SPT on topic structure sentences to facilitate global text processing. The results of this study showed that only L2 readers spent increased SPT on viewpoint sentences when moving from non-stress to stress conditions. This means that L2 readers tend to reread viewpoint sentences under stress situations. This is consistent with the hypothesis that readers tend to utilize the effective topic structure processing strategy to facilitate comprehension when their working memory resources were depleted by stress effects. For L1 readers, topic structure processing was not affected by stress situations.

The current study found that L1 readers used significantly more FPT and significantly less SPT on topic-introducing sentences (TS) than L2 readers. This means that L1 readers are more sensitive to topic structures than L2 readers and that they are more proficient at utilizing topic structure sentences to help construct global text representation. Interestingly, readers paid different levels of attention on topic structure sentences and viewpoint sentences. L1 readers paid more attention to topic structure sentences, while L2 readers focused more on viewpoint sentences. This may be related to the amount of information conveyed by the topic sentences of specific articles. In the texts used in this experiment, topic structure sentences are all shorter sentences and only play the role of introducing topics, while viewpoint sentences contain a larger amount of information. Therefore, when L2 readers find it difficult to construct a situational model of overall text under stress situations, they tend to adopt the strategy of reanalyzing VS to facilitate understanding. Since TS is more recognizable than VS in the provided reading materials, L1 readers may be more aware of the identity of TS and spend more time on reading it. But, because L1 readers’ global text processing is not affected by stress, they do not need to reanalyze topic structure sentences to enhance comprehension.

In sum, for L2 readers, stress showed an adverse effect on syntactic parsing, sentence integration, and global text processing. Specifically, L2 readers tend to facilitate global text processing by paying more attention to the topic structure of the text, while, for L1 readers, only the efficiency of syntactic parsing and sentence integration was affected by stress, and thus their global text processing capability was not affected. This is probably because reading is a highly automatized task for L1 readers, and the compensatory strategy they use is sufficient for them to process sentences and build situational models efficiently.

### 5.3. How Trait Anxiety and the Central Executive Function Interact with Stress Situations in Affecting Higher-Level Text Processing (Q3)

We examined the relationship between text processing (especially topic structure processing), reading performance, and personal characteristics in terms of trait anxiety, the central executive, and self-reported reading proficiency among L1 and L2 groups.

#### 5.3.1. Examination on L1 Group

The relationships between text processing and reading performance were first investigated for L1 readers. The only text processing measurement that showed a correlation with performance was BtSR. Those L1 readers who exhibited more between-sentence regressions demonstrated higher reading accuracy and efficiency. Moreover, topic structure processing measurements of L1 readers were only related with reading accuracy. L1 readers who spent less time on rereading viewpoint sentences had higher reading accuracy. Typically, readers who already constructed good topic structures would speed up the rereading process of key sentences. So, the increased reanalysis of viewpoint sentences indicates the difficulties readers faced during reading comprehension task. It can be inferred that L1 readers who faced challenges in constructing topic structures tended to have lower reading accuracy.

The relationships between text processing and personal characteristics were also investigated for L1 readers. Trait anxiety was the only personal characteristic that was found to be correlated with text processing measures. The results showed that those L1 readers with high trait anxiety demonstrated greater WtSR, FPT, FPRT, and RPRT. This result is consistent with the ACT in that stress has stronger negative effects on those readers prone to anxiety. Anxiety consumes working memory resources, leaving insufficient resources for text processing. Thus, those highly anxious L1 readers increased reading time and made more regressions to compensate for decreased processing efficiency. Moreover, topic structure processing measures were found to be correlated with self-rated reading proficiency. Those L1 readers who rated themselves with high reading proficiency spent more time on topic sentences during first-pass reading. This result may be explained by the fact that skilled readers are sensitive to topic structures and process topic sentences more slowly than other sentences that elaborate established discourse topics when they first encounter topic sentences. The result indeed showed that L1 readers with high English proficiency paid more attention to topic structure and were more experienced in processing topic structures.

#### 5.3.2. Examination on L2 Group

For L2 readers, we found that their performance did not show any correlations with text processing measurements, but was related with topic structure processing measurements. L2’s reading accuracy was positively correlated with the SPT spent on TS, such that L2 readers who spent more time on rereading topic structure sentences had higher reading accuracy. This result showed that the reanalysis of topic-introducing sentences can assist in understanding the text.

The eye-movement measures that represent text processing had been found to be related with the central executive and self-report reading proficiency among L2 readers. In the L2 group, the central executive was found to be related with reading proficiency. L2 readers who reported having more executive problems rated themselves with higher reading proficiency. L2 readers with higher reading proficiency made less regressions both within and between sentences, which indicates that they faced less difficulties during text processing. Additionally, topic processing measurements were also related with personal characteristics in the L2 group. L2 readers with more executive problems paid less attention to viewpoint sentences when they first encountered text. The deficiency in central executive impedes readers to adopt an effective topic processing strategy. The results also showed that L2 readers with high reading proficiency looked back to topic sentences more frequently. This result again supports the previous finding that referring back to topic sentences is an effective topic processing strategy to promote the construction of the overall representation of the text [45].

### 5.4. Further Discussion

In general, reading performance (i.e., the accuracy and efficiency of answering comprehension questions) is more associated with global processing, especially the process of topic structure processing, rather than the syntactic parsing process. This highlights the importance of constructing topic structure representation when conducting reading comprehension tasks.

One interesting finding was that those L1 readers who showed signs of deficiency in topic processing (e.g., reanalysis of topic sentences) had lower reading performance (accuracy), while those L2 readers who spent more time on rereading topic sentences had better performance. This is probably because those proficient L1 readers update a representation of the text’s topic structure each time they encounter topic sentences, so they can construct good topic structures efficiently during first-pass reading without the need to reanalyze topic sentences, while those less proficient readers had difficulties in constructing overall the topic structure during first-pass reading and require additional effort to reanalyze topic structure. But, for the L2 group, the current reading tasks generally put a greater strain on central executive resources in L2 than in L1, so L2 readers generally faced challenges in constructing topic structure. Only those of the L2 group who noted the importance of topic structure processing and insisted on constructing topic structure had a better understanding of the text and achieved higher reading performance than those who took no notice of topic structure processing.

Contrary to expectations, this study did not find significant interaction effects between stress situations and trait anxiety or between stress situations and the central executive on both text processing and topic processing measures. Trait anxiety and central executive functions both showed significant effects on some text processing measures. A possible explanation for this might be that the reading tasks were designed to be difficult enough to induce stress on those readers prone to anxiety, even in sessions without stress manipulations. In sum, L1 readers’ higher-level text processing was majorly affected by trait anxiety, while L2 readers’ text processing was majorly related to their reading proficiency. Self-rated reading proficiency was found to be related to topic processing in both L1 and L2 groups. Readers with higher reading proficiency showed better processing ability to build topic structures. This result highlights the importance of topic structure processing in reading comprehension tasks.

In the above discussion, the effects of the stress manipulations in session 2 and session 3 were not discussed separately; both session 2 and session 3 were considered as stress conditions. This is because, for most outcome variables, major differences were only found between session 1 (i.e., no stress condition) and the other two sessions—either one of them or both. Since the stress states of participants in each session were not assessed during the experiment, the stress effects induced by different manipulations cannot be quantified. But there are indeed some differences between the stress effects induced in session 2 and session 3. For example, the BtSR of L2 readers decreased from session 1 to session 2, but then increased substantially in session 3. However, it would be hard to distinguish whether the increased BtSR was caused by a stronger stress effect or by readers’ adjustment to the reading strategy. For further improvements of this work, future studies should assess the subjective states of participants, and readers’ adaptation to stress situations should be investigated.

When examining stress’s effect on cognitive performance, previous studies usually assessed performance in two regards, namely effectiveness and efficiency. Previous studies primarily used the accuracy and reaction time of answering questions to represent effectiveness and efficiency. But, in the current study, the effectiveness and efficiency of text processing cannot be assessed directly. This is because in the experiment, participants were presented with text first and were asked to comprehend the passage before answering questions, and then the questions were shown together with the text and readers were allowed to review the text again. So, the performance of readers’ responses to questions might include twice as much processing of the text. We noted that prior studies usually presented questions without showing texts. But, in the current experiment, we used difficult reading materials to produce stronger effects of stress on L1 reading performance, and so it would be incredibly difficult for the L2 group to answer questions without being shown text. Nevertheless, the performance of responding to questions can reflect the effectiveness and efficiency of text processing to some extent, as readers who can effectively and efficiently build good text representation during text processing do not need to reread the text when answering questions.

## 6. Conclusions

The present study adopted an eye-tracking approach to examine the effects of stress on higher-level text processing, especially focusing on the process of constructing topic structures, in L1 and L2 readers. The first key finding showed that stress negatively influenced syntactic parsing and sentence integration (evidenced by increased FPRT and RPRT) in both L1 and L2 readers. However, the impact of stress on global text processing was only observed in L2 readers (indicated by increased BtSR and VS_SPT). The second major finding was that under stress, L2 readers faced more difficulties in constructing topic structure (indicated by increased VS_SPT) compared to L1 readers. Moreover, this study revealed that L1 readers’ text processing can be affected by trait anxiety, while L2 readers’ processing was more related to their language proficiency. This study also confirmed the critical role of topic structure processing in building an efficient reading strategy.

These results contribute to both theoretical and applied implications for computer-assisted learning, providing empirical evidence based on eye-movement data. From a theoretical perspective, the findings advance our understanding of stress’s impact on different stages of higher-level text processing and may provide insights into the underlying mechanisms of stress’s influence on cognitive processing. Furthermore, this study highlights the necessity of taking moderating factors into consideration when examining stress’s effect on cognitive processing. In the context of reading, it is important to consider language proficiency, trait anxiety, and the central executive function in order to fully understand the effects of stress on reading.

From an applied perspective, these results can inform teaching practices by encouraging educators to guide L2 readers to focus more on topic sentences, especially in stress situations (e.g., tests), thereby optimizing their reading performance. These findings may have practical implications for developing technology-based interventions to help language learners suffering from stress disorders. It can also be useful in helping dyslectics by addressing accompanying emotional factors that make reading even more difficult for them. Furthermore, since the eye-tracking technique was adopted to record visual information during text processing, the findings of this work can provide insights into the design of eye-movement-based stress indicators, leading to instructional tools that support computer-assisted learning and assessment.

## 7. Limitation and Future Works

Some limitations of the current study and suggestions for future work should be noted. First, the current study primarily investigated text processing in paragraph reading, with reading materials comprising a single paragraph on one topic. Future work can consider using multiple-topic passages with a hierarchical organization as material to investigate more complex cognitive processes. Second, this study did not assess the participants’ perceived stress across different sessions, which limited the ability to differentiate the effects of varied stress manipulations. Future studies may consider assessing emotional, motivational, and cognitive perspectives of mental states in the experiment design for further examination. Third, as this study represents an initial investigation with a controlled and balanced design, we employed repeated measures ANOVA for its clarity and interpretability. Future research could employ more complex modeling approaches, such as linear mixed models (LMMs), to further analyze individual differences (e.g., cognitive ability, reading experience) and account for hierarchical data structures, such as longitudinal data, providing a more flexible framework for capturing inter-individual variability.

## Figures and Tables

**Table 1:** Eye-movement measures and their description.

Measure	Unit	Description
First-pass fixation time (FPT)	ms	Summed duration of all fixations on the target region before exiting it
First-pass rereading time (FPRT)	ms	Summed duration of all reinspective fixations on the target sentence during its first-pass reading
Regression path reading time (RPRT)	ms	The time from when the first fixation is made on the target sentence to when the fixation is made on the next sentence (including regressions back from current sentence previous sentences)
Second-pass fixation time (SPT)	ms	Duration of all fixations that were made on the target region after a reader had already fixated to the right of that region
Within-sentence regression (WtSR)	count	Any reinspective fixation on the target sentence
Between-sentence regression (BtSR)	count	Any fixation on text prior to the most recently fixated target sentence, including backward and forward fixations, as long as they do not return to the target sentence
TS_FPT, VS_FPT		The ratio of the FPT of topic structure sentences/viewpoint sentences (TS, VS) to the FPT of all sentences
TS_SPT, VS_SPT		The ratio of the SPT of topic structure sentences/viewpoint sentences (TS, VS) to the SPT of all sentences

**Table 2:** Descriptive statistics of eye-movement measures across stress sessions and language groups.

Measures	Session 1	Session 2	Session 3	L1	L2
WtSR	16.31 (11.46)	17.56 (10.37)	17.55 (10.46)	13.92 (8.81)	17.21 (10.60)
BtSR	6.49 (3.25)	6.46 (2.87)	6.71 (4.08)	6.32 (3.32)	6.91 (3.78)
FPT	7701 (3902)	8594 (3937)	7916 (3982)	6378 (2734)	8087 (3909)
SPT	63,881 (42,806)	67,710 (35,307)	69,411 (41,222)	53,580 (31,743)	67,907 (39,660)
FPRT	1695 (885)	2093 (1147)	1834 (1047)	1620 (912)	1998 (1174)
RPRT	8680 (3876)	10,286 (4843)	10,059 (5151)	7648 (3846)	9692 (4865)
TS_FPT	0.1721 (0.0497)	0.1719 (0.0522)	0.1903 (0.0569)	0.1883 (0.0477)	0.1787 (0.0537)
VS_FPT	0.3563 (0.0660)	0.3451 (0.0685)	0.3411 (0.0476)	0.3425 (0.0592)	0.3511 (0.0646)
TS_SPT	0.1279 (0.0323)	0.1275 (0.0306)	0.1282 (0.0320)	0.1267 (0.0310)	0.1279 (0.0312)
VS_SPT	0.4428 (0.0596)	0.4667 (0.0669)	0.4626 (0.678)	0.4573 (0.0638)	0.4538 (0.0634)

Note: Values are presented as mean (standard deviation).

**Table 3:** The results of repeated ANOVA on eye-movement measures.

Measures	Languages				Sessions				Languages × Sessions			
	**df**	F	***p*** **-Valuse**	$\eta^{2}$	**df**	F	***p*** **-Valuse**	$\eta^{2}$	**df**	F	***p*** **-Valuse**	$\eta^{2}$
Within-sentence Regression (WtSR)	1	32.356	0.000 ***	0.447	2	0.729	0.486	0.018	2	1.547	0.219	0.037
Between-sentence Regression (BtSR)	1	2.196	0.147	0.056	2	0.304	0.739	0.008	2	4.868	0.013 *	0.116
First-pass Fixation Time (FPT)	1	48.563	0.000 ***	0.542	2	2.543	0.089	0.058	2	0.044	0.949	0.001
Second-pass Fixation Time (SPT)	1	57.981	0.000 ***	0.604	2	0.759	0.442	0.020	2	2.294	0.121	0.057
First-pass Rereading Time (FPRT)	1	20.489	0.000 ***	0.350	2	5.667	0.006 **	0.130	2	0.597	0.542	0.015
Regression Path Reading Time (RPRT)	1	50.974	0.000 ***	0.600	2	5.587	0.008 **	0.141	2	3.033	0.063	0.082
ratio of FPT on topic sentences (TS_FPT)	1	9.922	0.003 **	0.211	2	1.928	0.154	0.050	2	0.275	0.756	0.007
ratio of FPT on viewpoint sentences (VS_FPT)	1	1.529	0.224	0.040	2	0.552	0.559	0.015	2	2.195	0.125	0.056
ratio of SPT on topic sentences (TS_SPT)	1	9.428	0.004 **	0.187	2	0.003	0.997	0.000	2	0.072	0.930	0.002
ratio of SPT on viewpoint sentences (VS_SPT)	1	3.145	0.085	0.080	2	1.696	0.191	0.045	2	3.405	0.043 *	0.086

* *p* < 0.05. ** *p* < 0.01. *** *p* < 0.001.

**Table 4:** Post hoc pairwise comparisons for the main effect of language.

Measures	(I) Language	(J) Language	Mean Difference (I-J)	SD	t-Value	*p*-Value	Cohen’s *d*
Within-sentence regression (WtSR)	L2	L1	12.958	2.278	5.688	0.000	1.145
First-pass fixation Time (FPT)	L2	L1	5268.430	756.008	6.969	0.000	1.784
Second-pass fixation time (SPT)	L2	L1	55,610.579	7303.202	7.615	0.000	1.805
First-pass rereading time (FPRT)	L2	L1	1091.995	241.245	4.526	0.000	1.439
Regression path reading time (RPRT)	L2	L1	6570.495	920.284	7.140	0.000	2.401
Ratio of FPT on topic sentences (TS_FPT)	L2	L1	−0.034	0.011	−3.091	0.003	0.704
Ratio of SPT on topic sentences (TS_SPT)	L2	L1	0.004	0.001	4.000	0.004	0.122

**Table 5:** Post hoc pairwise comparisons for the main effect of stress sessions.

Measures	(I) Session	(J) Session	Mean Difference (I-J)	SD	t-Value	*p*-Value	Cohen’s *d*
First-pass rereading time (FPRT)	Session 1	Session 2	−410.406	129.471	3.170	0.003	0.401
	Session 1	Session 3	−139.814	134.560	1.039	0.305	0.144
	Session 2	Session 3	270.592	105.933	2.554	0.015	0.246
Regression path reading time (RPRT)	Session 1	Session 2	−1574.369	576.168	2.732	0.010	0.359
	Session 1	Session 3	−1310.122	521.278	2.513	0.017	0.287
	Session 2	Session 3	264.247	399.590	0.661	0.513	0.053

**Table 6:** Comparisons of stress sessions using Helmert contrasts in repeated measures ANOVA.

Measures	Stress Session	df	F	*p*-Value
First-pass rereading time (FPRT)	Session 1 vs. Later	(1, 38)	5.174	0.029
	Session 2 vs. Session 3	(1, 38)	6.512	0.015
Regression path reading time (RPRT)	Session 1 vs. Later	(1, 34)	7.041	0.008
	Session 2 vs. Session 3	(1, 34)	0.437	0.513

**Table 7:** Correlations of measured variables for L1 readers.

Measures	Personal Variables			Reading Performance	
	1. Trait Anxiety	2. CE	3. Self-Rated Reading Proficiency	4. Accuracy	5. Efficiency
Within-sentence regression (WtSR)	0.440 **	0.058	0.062	0.191	0.137
Between-sentence regression (BtSR)	0.132	−0.159	0.213	0.337 **	0.279 *
First-pass fixation time (FPT)	0.313 *	0.112	−0.19	−0.02	−0.065
Second-pass fixation time (SPT)	0.299 *	0.134	0.07	0.168	0.15
First-pass rereading time (FPRT)	0.485 **	0.02	−0.173	0.072	0.035
Regression path reading time (RPRT)	0.321 *	0.139	−0.161	−0.041	−0.097
Ratio of FPT on topic sentences (TS_FPT)	0.006	0.008	0.251 *	0.008	0.033
Ratio of FPT on viewpoint sentences (VS_FPT)	−0.184	0.244	−0.186	−0.229	−0.212
Ratio of SPT on topic sentences (TS_SPT)	−0.024	0	−0.022	0.078	−0.084
Ratio of SPT on viewpoint sentences (VS_SPT)	0.028	−0.014	−0.019	−0.265 *	−0.221
1. Trait anxiety	1	0.057	−0.098	0.165	0.02
2. Central executive (CE)		1	−0.175	−0.388 **	−0.231
3. Self-rated reading proficiency			1	0.13	0.106
4. Accuracy				1	0.796 **
5. Efficiency					1

** Correlation is significant at the 0.01 level (2-tailed). * Correlation is significant at the 0.05 level (2-tailed).

**Table 8:** Correlations of measured variables for L2 readers.

Measures	Personal Variables			Reading Performance	
	1. Trait Anxiety	2. CE	3. Self-Rated Reading Proficiency	4. Accuracy	5. Efficiency
Within-sentence regression (WtSR)	0.164	−0.188	−0.297 *	−0.085	−0.213
Between-sentence regression (BtSR)	−0.082	−0.19	−0.289 *	−0.078	−0.255
First-pass fixation time (FPT)	0.072	−0.227	−0.064	0.02	−0.101
Second-pass fixation time (SPT)	0.031	−0.338 **	−0.111	0.002	−0.198
First-pass rereading time (FPRT)	−0.011	−0.131	−0.031	0.149	0.056
Regression path reading time (RPRT)	0.105	−0.315 *	−0.343 **	0.091	−0.209
Ratio of FPT on topic sentences (TS_FPT)	−0.121	0.007	0.211	−0.008	0.1
Ratio of FPT on viewpoint sentences (VS_FPT)	0.127	−0.274 *	−0.214	0.065	−0.175
Ratio of SPT on topic sentences (TS_SPT)	0.009	0.006	−0.012	0.258 *	0.103
Ratio of SPT on viewpoint sentences (VS_SPT)	−0.041	0.095	0.038	−0.049	0.109
1. Trait anxiety	1	0.322 *	−0.205	−0.167	−0.298 *
2. Central executive (CE)		1	0.348 **	−0.133	0.035
3. Self-rated reading proficiency			1	0.094	0.294 *
4. Accuracy				1	0.664 **
5. Efficiency					1

** Correlation is significant at the 0.01 level (2-tailed). * Correlation is significant at the 0.05 level (2-tailed).

## Data Availability

The raw data supporting the conclusions of this article will be made available by the authors on request.

## Abbreviations

The following abbreviations are used in this manuscript:

ACT	Attentional Control Theory
BtSR	Between-sentence Regression
CE	Central Executive
CTAS	Cognitive Test Anxiety Scale
FPT	First-pass Fixation Time
FPRT	First-pass Rereading Time
L1	First-language
L2	Second-language
RPRT	Regression Path Reading Time
SPT	Second-pass Fixation Time
TS	Topic Structure Sentence
VS	Viewpoint Sentence
WtSR	Within-sentence Regression
